# The Use of the Direct Optimized Probabilistic Calculation Method in Design of Bolt Reinforcement for Underground and Mining Workings

**DOI:** 10.1155/2013/267593

**Published:** 2013-07-02

**Authors:** Martin Krejsa, Petr Janas, Işık Yilmaz, Marian Marschalko, Tomas Bouchal

**Affiliations:** ^1^Department of Structural Mechanics, Faculty of Civil Engineering, VŠB-Technical University of Ostrava, 17 Listopadu 15, 708 33 Ostrava, Czech Republic; ^2^Department of Geological Engineering, Faculty of Engineering, Cumhuriyet University, 58140 Sivas, Turkey; ^3^Faculty of Mining and Geology, Institute of Geological Engineering, VŠB-Technical University of Ostrava, 17 Listopadu 15, 708 33 Ostrava, Czech Republic; ^4^Department of Environmental Engineering, Faculty of Mining and Geology, VŠB-Technical University of Ostrava, 17 listopadu 15, 708 33 Ostrava, Czech Republic

## Abstract

The load-carrying system of each construction should fulfill several conditions which represent reliable criteria in the assessment procedure. It is the theory of structural reliability which determines probability of keeping required properties of constructions. Using this theory, it is possible to apply probabilistic computations based on the probability theory and mathematic statistics. Development of those methods has become more and more popular; it is used, in particular, in designs of load-carrying structures with the required level or reliability when at least some input variables in the design are random. The objective of this paper is to indicate the current scope which might be covered by the new method—Direct Optimized Probabilistic Calculation (*DOProC*) in assessments of reliability of load-carrying structures. *DOProC* uses a purely numerical approach without any simulation techniques. This provides more accurate solutions to probabilistic tasks, and, in some cases, such approach results in considerably faster completion of computations. *DOProC* can be used to solve efficiently a number of probabilistic computations. A very good sphere of application for *DOProC* is the assessment of the bolt reinforcement in the underground and mining workings. For the purposes above, a special software application—“Anchor”—has been developed.

## 1. Introduction

The designing process, assessment of the reliability, and individual stages of production, assembly, or operation of the underground structure are affected now by many uncertainties which influence reliability of such constructions by its random nature which cannot be neglected. This means that the design and implementation processes start being affected by variability of features of the buildings and facilities.

It is possible to apply various calculation procedures based on the probability theory and mathematic statistics in designs and assessment of the reliability, this approach being more and more popular now. The key feature of the probabilistic method is that it is possible to express variability of input quantities in a stochastic (probabilistic) form, for instance, by histograms. Unlike the applicable standards and procedures which are based on deterministic expression of input quantities (using a single value—a constant), the probabilistic methods provide more precise reliability assessment and improved safety for those who use the buildings and structures.

## 2. Interpreting Random Quantities in Probabilistic Calculations

**Figure 1:**
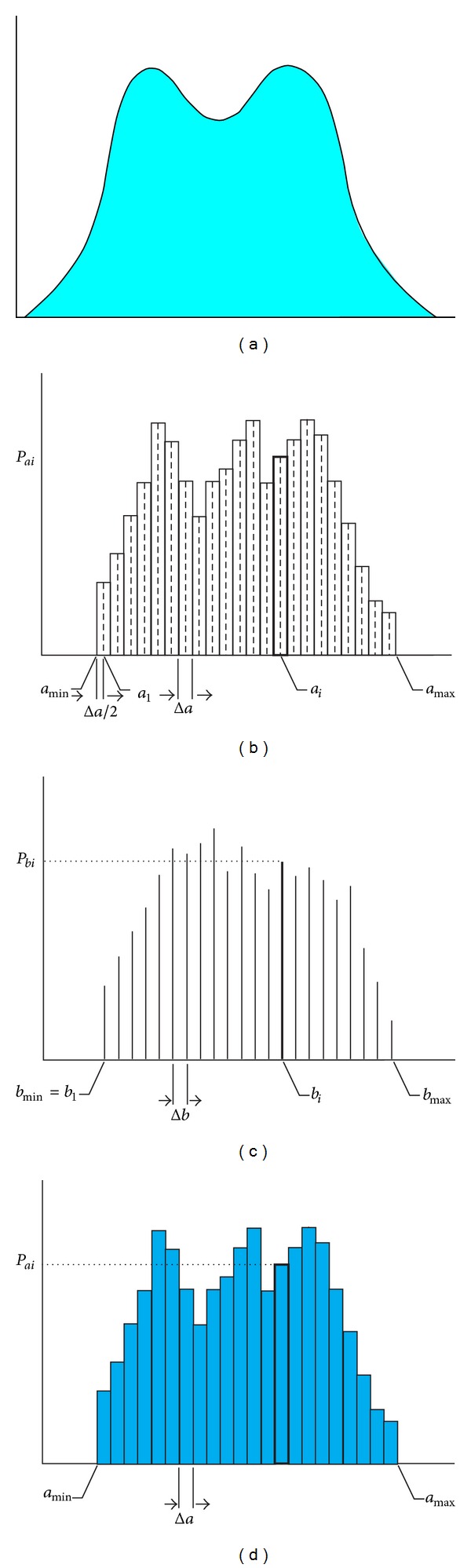
Approximation of the restricted probability distributions: (a) original approximation, (b) discrete approximation, (c) pure discrete approximation, and (d) piecewise uniform approximation.

Histograms which are a part of the calculation in probability tasks should be regarded as approximation of the original distribution of probability of the random quantity (Figure 1(a)). If the distribution inside the histogram classes is even, such approximation is the approximation of the original distribution of random quantity probability by even parts (Figure 1(d)). If the histogram classes are represented by only one value, the original distribution of the random quantity probability is approximated by discrete distribution of the probabilities (Figures 1(b) and 1(c)) which are used in *DOProC* calculations.

## 3. Function of Random Quantities

In probabilistic calculations, the calculation model defines a function with generally *n* random quantities *X*_1_, *X*_2_,…, *X*_*n*_. The resulting quantity—*Z* is expressed generally as follows:

(1) $$\begin{array}{c} Z=f\left(X_{1},X_{2},\ldots ,X_{n}\right). \end{array}$$

It is also a random quantity which can be expressed by statistic moments, parametric distribution, or empirical distribution of probability using a nonparametrically defined histogram.

## 4. Reliability of the Supporting Construction

During the construction design process, several computation operations are carried out with respect to the reliability assessment of specific structural part or the construction as a whole [1]. Various reliability criteria resulting from standards in force should be fulfilled.

**Figure 2:**
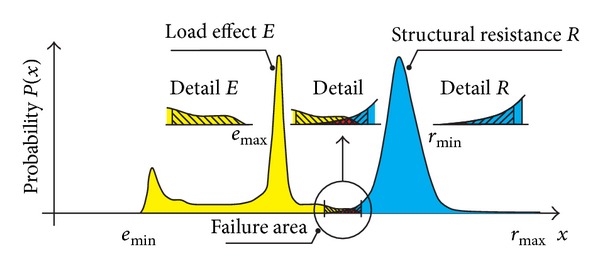
Probability density curves—load effect, *E*, structural resistance, *R*, and the area where a failure may occur.

The construction should be designed in such as way so that the structural resistance, *R*, would be higher than the load effects, *E*. Considering all random phenomena in the load, manufacturing and installation inaccuracies and inaccuracies where the construction is used, the structural resistance, *R*, and load effect, *E*, should be regarded as random quantities (Figure 2).

The probabilistic reliability assessment is based on the reliability condition which can be expressed as follows:

(2) $$\begin{array}{c} R-E\geq 0, \end{array}$$

where *R* is the structural resistance and *E* is the load effect. The left side of (2) is referred to as the reliability function, RF. Sometimes, it is also referred to as a failure function, *G*, or reliability reserve, *Z*. If the reliability condition (2) is not fulfilled, such situation is undesirable in terms of reliability—it is a failure when the load effect, *E*, exceeds the magnitude of the structural reliability, *R*. The area where a failure may occur is shown in Figure 2.

In the area where the histograms for the structural resistance, *R*, and load effect, *E*, overlap in Figure 2, it is possible to determine the failure probability, *P*_*f*_:

(3) $$\begin{array}{c} P_{f}=P\left(\text{RF}<0\right)=P\left(R-S<0\right). \end{array}$$

**Figure 3:**
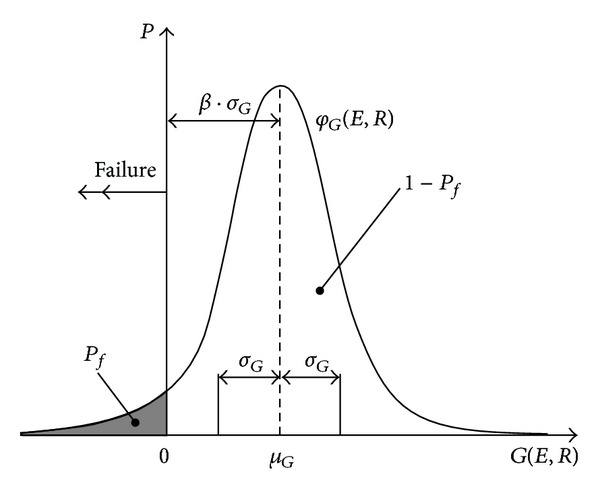
Determining the failure probability, *P*_*f*_, and reliability index, *β*, by means of the failure reliability, RF (the failure function, *G*).

The magnitude of the failure probability is influenced by the negative part of the RF histogram. The nonfailure probability, *P*_*s*_, equals 1 − *P*_*f*_ (see, e.g., Figure 3).

The estimated failure probability, *P*_*f*_, with respect to the reliability condition is defined by [2]

(4) Pf=P(R−S<0)=∫Dff(X1,X2,…,Xn)dX1,dX2,…,dXn,

where *D*_*f*_ is the failure area and RF < 0; a *f*(*X*_1_, *X*_2_,…, *X*_*n*_) is the function of combined probability density for random quantities *X* = *X*_1_, *X*_2_,…, *X*_*n*_.

## 5. Designed Failure Probability

A degree of the structural reliability in the probabilistic calculation is the ultimate designed value of the failure probability, *P*_*d*_, (the designed probability) or the reliability index, *β*. The structure is reliable only if the following reliability condition is fulfilled:

(5) $$\begin{array}{c} P_{f}<P_{d}, \end{array}$$

(6) $$\begin{array}{c} \beta_{d}<\beta . \end{array}$$

The designed failure probability, *P*_*d*_, (or the reliability index, *β*) is determined on the basis of the required reliability level, type of the ultimate state, and estimated service life of the structure, *T*_*d*_. Reference values for the designed probabilities, *P*_*d*_, or reliability index, *β*, are specified in the European standards in force.

In order to differentiate the reliability, the following classes of consequences were introduced in Eurocodes CC1, CC2, and CC3 (where CC stands for consequences classes). Such consequence classes take into account consequences of failures or nonfunction incapacity of the construction. Reliability classes—RC1, RC2, and RC3—were defined on the basis of the reliability index, *β*. The reliability classes are related to the consequence classes CC1, CC2, and CC3.

Figure 3 shows the curve based on the definition of the reliability structure (2) with a normal distribution of probabilities for the structural resistance, *R*, and load effect, *E*. In accordance with (3), the failure occurs also if the failure function *G* < 0. The reliability index, *β*, is then the distance between the mean failure function, *G*, from the start defined in standard deviation units, *σ*_*G*_. For the reliability index, one obtains

(7) $$\begin{array}{c} \beta =\frac{\mu_{G}}{\sigma_{G}}, \end{array}$$

where the mean value, *μ*_*G*_, is the difference:

(8) $$\begin{array}{c} \mu_{G}=\mu_{R}-\mu_{S} \end{array}$$

and the standard deviation, *σ*_*G*_, is expressed by

(9) $$\begin{array}{c} \sigma_{G}=\sqrt{\sigma_{R}^{2}-\sigma_{E}^{2}}, \end{array}$$

where *μ*_*R*,*E*_ are respective mean values of the structural resistance, *R*, or load effects, *E*, and *σ*_*R*,*E*_, are the standard deviations for the structural resistance and load effect.

## 6. Using Probabilistic Methods for Random Variable Models

It is often very difficult to determine the failure probability, *P*_*f*_, on the basis of the explicit calculation of the integral (4). A number of stochastic methods have been, and are being, developed [5] to solve (4).

The most frequently used and most numerous group of the computational method comprises the simulation methods which are based on the popular simulation technique—Monte Carlo (Direct Sampling, e.g., Bjerager [6]) or any advanced or stratified simulation methods (Latin Hypercube Sampling, (LHS), Stratified Sampling, Importance Sampling, Adaptive Sampling, Bucher [7]) which estimate the failure probability, *P*_*f*_, using fewer simulations than the frequently used Monte Carlo.

Eurocodes which are in force now mention the application of approximation methods—First/Second Order Reliability Method (abbreviated to FORM and SORM, der Kiureghian and Dakessian [8]) which are used mostly for calibration of partial coefficients. These computational methods employ for approximation of the final reliability function (the failure) a simple approximation—typically, a normal distribution of the probability. The integral (4) is solved then analytically. The response surface method [9, 10] is one of the next approximation methods.

Both the original method and the new method which are under development now—the Direct Optimized Probabilistic Calculation (*DOProC*)—use a purely numerical approach and basics of the probabilistic calculation without any simulation techniques to solve (4). This provides more accurate solutions to probabilistic tasks, and results, in some cases, in considerably faster completion of computations.

## 7. Direct Optimized Probabilistic Calculation (*DOProC*)

The Direct Optimized Probabilistic Calculation (*DOProC*) has been developed since 2002. The original name of this method was the Direct Determined Fully Probabilistic Method (DDFPM). The word “Determined” in the name of the method means that the calculation procedure for a certain task is clearly determined by its algorithm, while Monte Carlo generates calculation data for simulation on a random basis. The name of the method was discussed and consulted with experts in the structural reliability, the conclusion being that the word “Determined” in the name of the method is somewhat misleading. Consequently, the name of the method was modified. The new term in the name of the method—“Optimized”—is based on the following facts. The number of variables that enter calculation of the failure probability, *P*_*f*_, computation is, however, limited by capabilities of the software to process the application numerically. If there are too many random variables, the application is extremely time demanding—even if high-performance computers are used.

The computational complexity of *DOProC* is given, in particular, by

- the number of random input quantities *i* = 1,2,…, *N*;
- the number of histogram classes (intervals) for each random input quantity, *n*_*i*_;
- complexity of the task (computational model),
- the probabilistic computation algorithm (the way used to define the computational model).

Therefore, efforts have been made to reduce the number of operations. The purpose of the *DOProC* optimizing techniques is to minimize the computing time since the algorithm is limited to a certain extent, in particular, for extensive applications where too many simulations exist. If the optimizing techniques are used in *DOProC*, the failure probability, *P*_*f*_, can be determined in a real time. On top of this, results are reliable and accurate enough even in relatively demanding probabilistic tasks.

The optimizing techniques include the following.

- *Grouping of variable input quantities* (such as load components) which may enter the calculation jointly and a joint histogram can be prepared in advance.
- *Interval optimizing* where the number of intervals of variable input quantities of individual histograms is decreased, while the whole range for each random input quantity is maintained.
- *Zone optimizing* where only intervals affecting a certain value, for instance, the failure probability of a structure, *P*_*f*_ are involved.
- *Trend optimizing* which considers the correct direction (trend) in the algorithm of the probabilistic calculation.
- *Grouping of partial calculation results*, for instance, in creation of the resulting reliability function, RF.
- *Computation parallelization* where the computation is carried out in several processors or cores at the same time.
- *Combination of the optimizing procedures* above.

For instance, Janas et al. [11] include detailed theoretical background for the *DOProC* algorithm including the optimizing procedures which make it possible to determine in the reliability assessment the failure probability, *P*_*f*_, for two or more random quantities. Currently, the *DOProC* along with the optimizing steps can address well several probabilistic tasks. It is possible to use ProbCalc in *DOProC*. ProbCalc is a software application which is still under development. It is rather easy and simple to implement quite a complicated analytical transformation model of a probabilistic task defined in a character form or as a dynamic DLL library similarly as in Tvedt [12], Thacker et al. [13], and Cervenka et al. [14]. A lite version of this software can be downloaded from the webpage http://www.fast.vsb.cz/popv/ [15].

## 8. Probabilistic Calculation of Reliability of Bolt Reinforcements

The probabilistic approach to the assessment and design of the structures has started appearing in practice recently only. These computational procedures are used, in particular, in designs of load-carrying systems for ground structures—for instance, for steel structures [16–18], for reinforced concrete structures [19–21], or other engineering activities [22]. For underground and mining workings, this approach is used in rare cases only.

The methods for the design of reinforcements in the underground workings were based, generally, on an assumption that the input values were clearly deterministic. This is the case not only of geological or technical conditions under which the bolts will be applied but also properties of the bolts that are influenced also by installation procedures. Most input data used in various design methods in connection with the bolts are random. When designing the underground workings, it is rather easy to use the deterministic approach. It, however, does not take into account the random nature of input quantities which, in turn, are almost neglected in the designing of the bolts.

**Figure 4:**
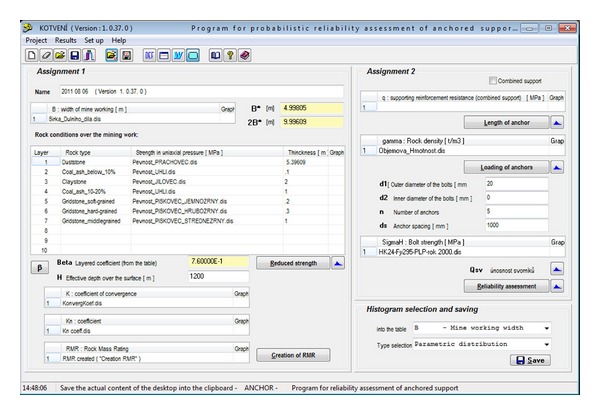
Desktop in the Anchor software [3].

It is just this area where the probabilistic (stochastic) method appears to be very efficient for determination of the necessary load-carrying capacity of the bolt reinforcement. That method represents an entirely new approach to this field. Most successful applications of the *DOProC* include guidelines for probabilistic designs and reliability assessment of underground and mining workings [23, 24] and creation of the software—Anchor (Janas et al. [3]; for the Anchor desktop see Figure 4).

When designing the bolt support for certain conditions, the following parameters need to be defined:

- the length of bolts;
- the number and location of the bolts near the mining working or underground working;
- parameters of the bolts (the type, diameters, material, anchoring method, etc.).

Extensive measurements were carried out in the mining workings in the Ostrava-Karviná Colliery. It follows from the measurements that the convergence, this means dislocation of rock into the mining working, can be calculated from the following formula:

(10) $$\begin{array}{c} u=0,1B\cdot \left(1-e^{-0,015t}\right)\cdot \left(e^{(1,2H-q)/45\sigma_{r}\;}-1\right), \end{array}$$

where *H* is the efficient depth under the surface (m), *B* the dimension (typically, the width) of the mining working [m], *t* is the time in days, *q* is the load-carrying capacity of the support [kNm^−2^], and *σ*_*r*_ is the reduced strength of the hanging rock [MPa] which is determined as follows:

(11) $$\begin{array}{c} \sigma_{r}=\beta \frac{\sum_{1}^{n}\sigma_{di}m_{i}}{2B}. \end{array}$$

In relation (10) *β* is the stratification coefficient pursuant (see Table 1), *σ*_*di*_ is the strength in one-axis compression of the *i*th strata, and *μ*_*i*_ is the thickness of the *i*th strata.

Nonelastic deformation range, *B*_*n*_, which is the basis for specification of loading and length of the bolt can be described, using (10) and for *t* → *∞*, as follows:

(12) $$\begin{array}{c} B_{n}=0,251189\cdot B\cdot K_{n}\cdot {\left(e^{(1,2H-q)/45\sigma_{r}\;}-1\right)}^{0,6}. \end{array}$$

*K*_*n*_ characterizes the relation between the nonelastic deformation in the mining working or under working with the *B* dimension, *B*_*n*_ convergence, and *σ*_*r*_ reduced strength. In past, a single one deterministic value was used in spite of the fact that this quantity is of a random nature.

The load to be transferred by the bolted support should be suitable for the nonelastic deformation range (*B*_*n*_), rock weight (*γ*) as well as for a certain level of self-bearing capacity of rock strata that does not exist in the nonelastic deformation range. Using the geomechanical classification parameter (RMR, rock mass rating) has proved to be a good solution [4]. Then, the load of the bolted support was determined by the following formula:

(13) Q=Bn·B·γ·100−RMR100  =2,51189B2γ100−RMR100Kn(e(1,2H−q)/45σr  −1)0,6,

where *γ*  is the specific gravity of rock [10^3^ kg·m^−3^] and *Q* is the total load of the bolted reinforcement per running meter in the working [kN].

The assessment of reliability of bolted reinforcements in underground and mining workings is based on the reliability function (RF) analysis pursuant to (2) that is described using the following formula:

(14) $$\begin{array}{c} \text{RF}=Q_{\text{sv}}-Q, \end{array}$$

where *Q*_sv_ is the load-carrying capacity of the bolts and *Q* is the bolt loading per running meter in the working. The load-carrying capacity of the bolts is based on the following formula:

(15) $$\begin{array}{c} Q_{\text{sv}}=n_{\text{sv}}q_{\text{sv}}=\frac{n\cdot q_{\text{sv}}}{d_{s}}=\frac{n\pi {\left(d_{1}-d_{2}\right)}^{2}\cdot \sigma_{\text{sv}}}{4d_{s}}, \end{array}$$

where *n*_sv_ is the total number of bolts per running meter in the working, *n* is the number of bolts in a row, typically, vertically to the working's axis, *q*_sv_ is the load-carrying capacity of one bolt, *d*_1_ is the bolt's outside diameter, *d*_2_ is the bolt's inside diameter, *d*_*s*_ is the span between the anchor rows, and *σ*_sv_ is the normal stress in one bolt.

In addition to the load and required load-carrying bolt reinforcement, the required length of the bolts is another important parameter which should correspond to the range of nonplastic deformations, *B*_*n*_, close to the underground or mining working. It follows from practical observations and measurements in mines that, if the bolt supports are installed, the convergence into the mining working is less that that calculated from (9) where the convergence is determined for the workings supported by bracing supports. The reason is that the resistance against dislocation of rock pillar appears only after the rock-support contact is established. This results in more extensive deformation of the rock pillars, if compared with the bolt reinforcement. Data resulting from the comparison of deformation in the workings supported by the bolt reinforcements and *u* in (9) can be used to calculate the length of bolts, *l*, in the hanging wall as follows:

(16) $$\begin{array}{c} l=0,251189\cdot K_{n}\cdot B\cdot K\cdot {\left(e^{(1,5H-q)/45\sigma }-1\right)}^{0,6}, \end{array}$$

where *K* is the set of values obtained from experiments. In spite of the fact that *K* is variable, it is, for working purposes, marked as a convergence coefficient.

Specific databases of the random input variables were used to create histograms of input quantities pursuant to 1.b. The basis was measurements done by manufacturers of the anchoring components and in mines where the bolt supports were installed. 

In the proposed methodical guideline, there are still some input variables that are expressed by deterministic description: stratification coefficient, *β*, efficient depth under the surface, *H*, thickness of individual strata, *m*_*i*_, outside and inside diameters of the bolts, *d*_1_ and *d*_2_, and distance between the bolt rows, *d*_*s*_.

## 9. Software for Calculations of Failure Probability of a Bolt Reinforcement

A *DOProC*-based software application named “Anchor” [3] was created for the probabilistic assessment of reliabilities of the bolt reinforcements used in the mining and underground workings. Using this software, it is possible to assess and design the bolt reinforcement very flexibly.

**Figure 5:**
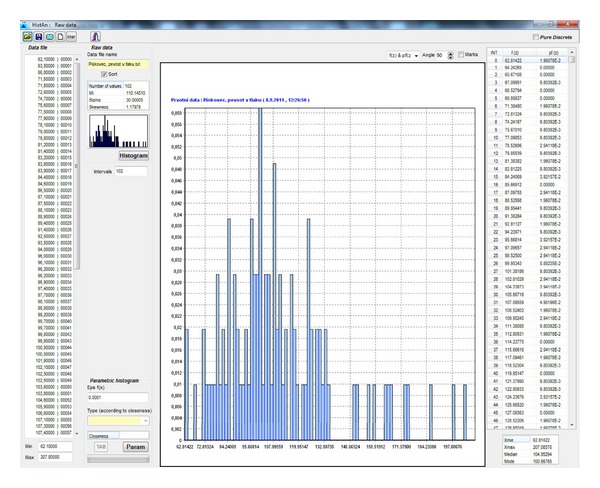
Histogram with empirical distribution of probabilities created from measured compression strength in carboniferous sandstone [MPa].

**Figure 6:**
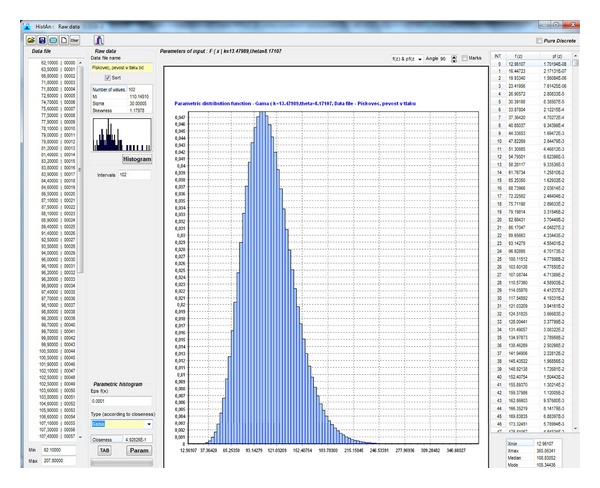
Histogram with parametric distribution of probabilities for compression strength in carboniferous sandstone [MPa].

Figure 4 shows the Anchor desktop with input parameters for the sample calculation. Using this software application, the measured data can be processed to create histograms and derive parameters. The best distribution is chosen from among of dozens of known parametric distributions on the basis of a coefficient that is referred to as a tightness coefficient.  Figure 5 shows the histogram of primary data prepared on the basis of 102 measurement data when the compression strength was measured in carboniferous sandstone. The horizontal axis shows the compression strength in MPa, while the vertical axis shows the probability of occurrence. The number of classes is equal to the number of primary data.  Figure 6 shows the assessment made by means of a histogram for parametric distribution of Gamma probability with the higher proximity coefficients. Such distribution is most creditworthy, from the point of view of statistics. If another type of parametric distribution or another number of classes is chosen, which is possible, the proximity coefficient will be smaller.

**Figure 7:**
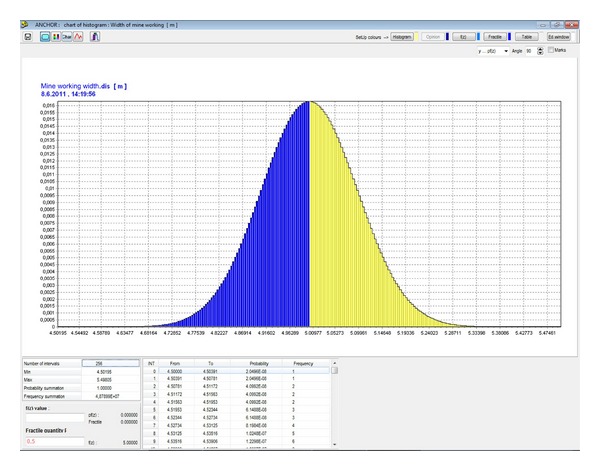
Histogram of the width of the mining working *B* [m].

**Figure 8:**
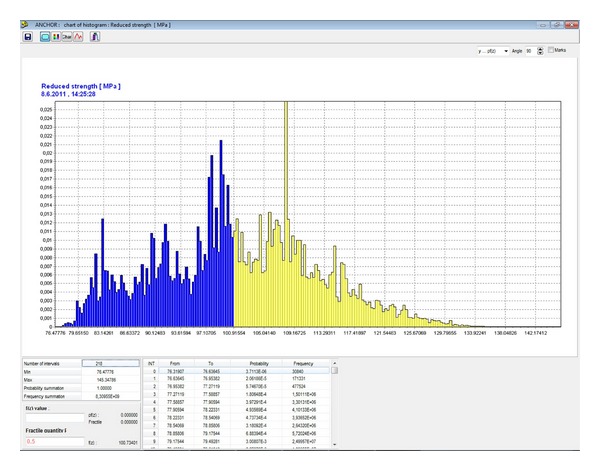
Histogram of the reduced strength of hanging rock *σ* [MPa].

**Figure 9:**
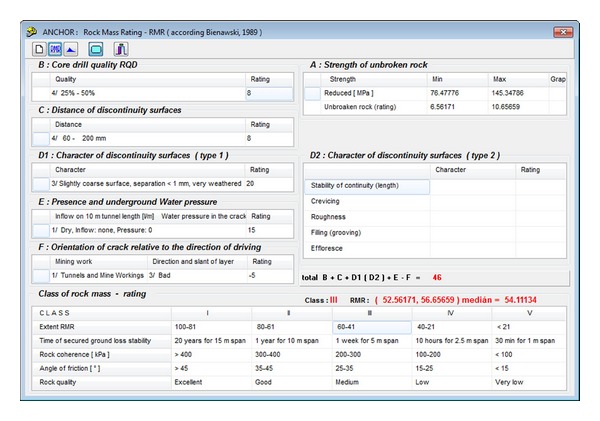
Software desktop with a table for determination of rock mass rating (the geomechanical classification coefficient RMR), [4].

**Figure 10:**
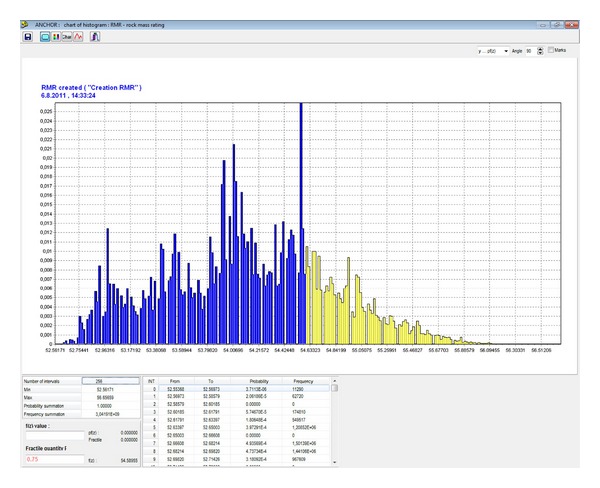
Histogram of rock mass rating (the geomechanical classification coefficient RMR) [4].

In the first stage of the probabilistic calculation, a histogram with parametric probability distribution (Figure 7) of a reduced strength of hanging rock, *σ*, is determined pursuant to (10) (Figure 8) for the specified *B* width of the mining working, for the specified composition and thickness of the stratum. This histogram is needed for determination of the length and the loading of the anchors and for the geomechanical classification coefficient RMR [4]. For that purpose, a separate table in the application is used (Figure 9). The result is the histogram for the geomechanical classification coefficient RMR—rock mass rating (Figure 10).

**Figure 11:**
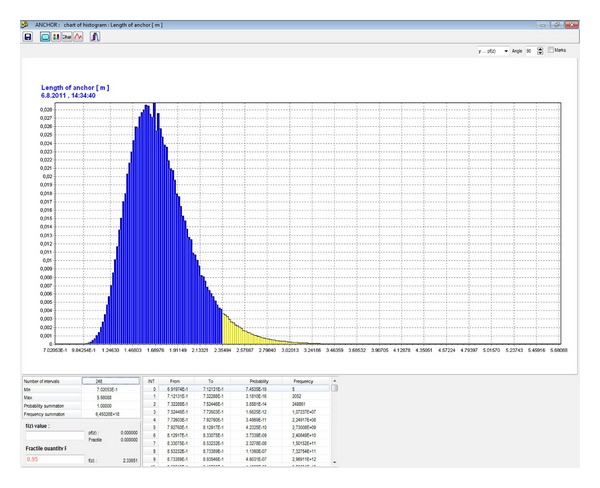
Histogram of the length of the designed bolt *l* [m].

Then, it is possible to determine a histogram for the length of the proposed bolt, *l*, pursuant to (15) (Figure 11). Using the histogram, it is possible to obtain the required length for the specific level of reliability.

**Figure 12:**
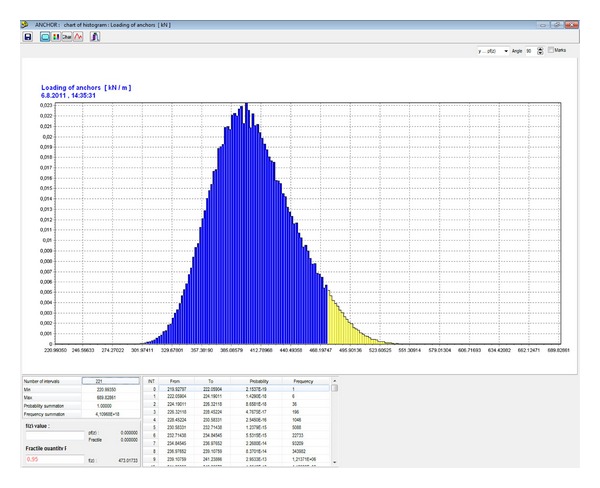
Histogram of the bolt load *Q* [kN/m].

**Figure 13:**
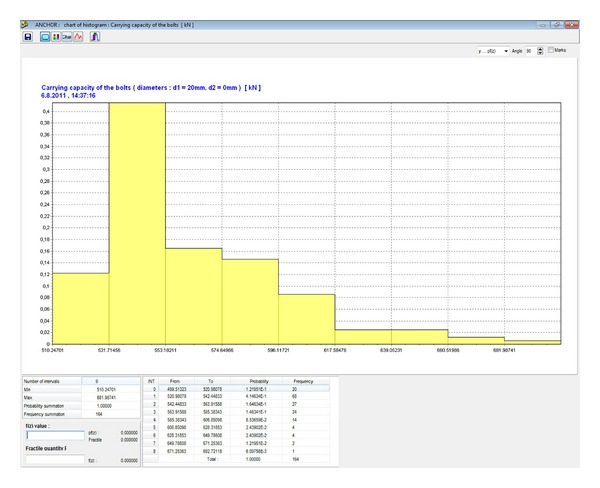
Histogram of the load-carrying capacity of the bolts *Q*_sv_ [kN].

**Figure 14:**
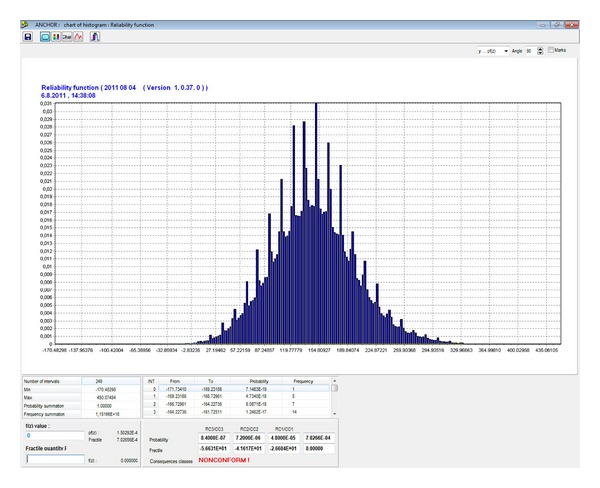
Histogram of the RF reliability function where the failure probability is *P*_*f*_ = 7.0266 · 10^−4^, for 5 bolts per one meter of the underground working.

In the final design of the bolt reinforcement, five steel bolts per running meter were chosen. The diameter of each bolt is 20 mm (see Figure 4). The calculated histograms of the load-carrying reliability of each bolt, *Q*_sv_, are included pursuant to (14) (see Figure 13) and bolt load, *Q*, pursuant (12) (see Figure 12) into the reliability function, RF, (13). The final failure probability, *P*_*f*_, of the working is determined, obtained from the analysis of the resulting RF histogram in Figure 14. The failure probability can be used to assess the reliability of the designed bolt reinforcement.

## 10. Final Assessment of Reliability of a Bolt Reinforcement

The final probabilistic failure was specified as *P*_*f*_ = 7.0266 · 10^−4^ for the designed bolt reinforcement of the underground or mining working. Considering the stringent reliability criteria for the mining workings which are in force, for instance, in *EN 1990*, the bolt reinforcement would not meet the requirements—the design probability, *P*_*d*_, for RC1 (minor consequences) is 4.8 · 10^−4^ in the standard. This means that the reliability condition (5) is not fulfilled. In this case, a solution would be to increase the number of bolts or to increase the diameter of bolts. An open issue is still the permitted failure probability, *P*_*d*_, of reinforcements used in the underground and mining workings.

## 11. Conclusions

This paper discusses development of probabilistic methods and application of the probabilistic methods in assessment of reliabilities of underground and mining workings. Using the proposed method, it is possible to apply probability calculations in the designing and assessment of reliability of the bolt reinforcement installed in mining and underground workings. Thus, it is possible to determine the length and load-carrying capacity of the bolts. The prerequisite is, however, a sufficient database of input quantities including the experience from practical operation because many input quantities cannot be based on models and laboratory measurements only.

The probabilistic approach which has been described above for the underground and mining reinforcement as well as the available database of histograms for random input variables can be used for other structures and methods for calculation of underground and mining constructions.

The proposed guidelines are based on the original approach as well as on the new methods—Direct Optimized Probabilistic Calculation (*DOProC*)—which is still under development. *DOProC* appears to be a very efficient tool that provides a solution which is affected by a numerical error and by an error resulting from the discretising of the input and output quantities, only *DOProC* is well suited for various probabilistic tasks. A lite version of the software which has been developed specifically for the probabilistic design and assessment of the bolt reinforcement can be downloaded from the website http://www.fast.vsb.cz/popv/ [15]. Using this software, probabilistic calculations can be solved very flexibly in a real time.

## Figures and Tables

**Table 1:** *β* stratification coefficient.

Number of strata	1	2	3	4	5	6	7	8	9	10
*β*	1.0	0.95	0.90	0.86	0.82	0.79	0.76	0.73	0.71	0.70
